# Circulating soluble receptor of advanced glycation end product is associated with bicuspid aortic aneurysm progression via NF-κB pathway

**DOI:** 10.1093/icvts/ivab242

**Published:** 2021-10-14

**Authors:** Hao Jia, Le Kang, Shuyang Lu, Zhenhang Chen, Jinqiang Shen, Ben Huang, Yunzeng Zou, Yongxin Sun

**Affiliations:** Department of Cardiac Surgery, Zhongshan Hospital, Fudan University, Shanghai, China; Department of Cardiac Surgery, Zhongshan Hospital, Fudan University, Shanghai, China; Department of Cardiac Surgery, Zhongshan Hospital, Fudan University, Shanghai, China; Department of Cardiac Surgery, Zhongshan Hospital, Fudan University, Shanghai, China; Department of Cardiac Surgery, Zhongshan Hospital, Fudan University, Shanghai, China; Department of Cardiac Surgery, Zhongshan Hospital, Fudan University, Shanghai, China; Central Laboratory of Cardiovascular Institute, Zhongshan Hospital, Fudan University, Shanghai, China; Department of Cardiac Surgery, Zhongshan Hospital, Fudan University, Shanghai, China

**Keywords:** Bicuspid aortic valve, Circulating sRAGE, NF-κB pathway, Biomarker, Aortic aneurysm

## Abstract

**OBJECTIVES:**

Patients with bicuspid aortic valve (BAV) have a high risk of aortic dilation and adverse vascular events. Previous studies had reported soluble receptor for advanced glycation end products (sRAGE) to compete with receptor of advanced glycation end products (RAGE) for ligand binding and inhibit the activation of nuclear-factor kappa-B (NF-κB) pathway and matrix metalloproteinases (MMP) transcription. Thus, sRAGE serum levels may contribute to the clinical diagnosis and monitoring of ascending aorta aneurysm in patients with BAV.

**METHODS:**

To eliminate the confounding factors, 44 patients with BAV were divided into 3 subgroups according to the diameter of ascending aorta, and 20 patients with tricuspid aortic valve and normal-sized ascending aorta were selected as a control group. Protein levels and gene transcription of several variates were evaluated in the tissue and serum samples from these patients. Human aortic smooth muscle cells were treated with AGE-BSA in gradient concentrations, and changes in phenotype and protein and mRNA levels were detected.

**RESULTS:**

Serum levels of sRAGE in the 3 BAV groups were obviously higher than those in the tricuspid aortic valve group, although there was negative correlation between the serum sRAGE levels and ascending aortic diameters among patients with BAV. Transcript expression levels of RAGE and NF-κBp65 mRNA were increased in the 3 BAV groups and RAGE/NF-κB pathway was activated with the progression of ascending aortic aneurysm. Abnormal activation of RAGE/NF-κB pathway was observed in AGE-BSA-treated human aortic smooth muscle cells.

**CONCLUSIONS:**

Our study has shown a trend in serum levels of sRAGE among patients with BAV, and that the cellular and extracellular pathological processes are quite serious even in the normal-sized or slightly dilated aorta. Together, the findings indicated that sRAGE may be used as a biomarker to predict aneurysm expansion rates and the risk of adverse vascular events.

**Figure:**
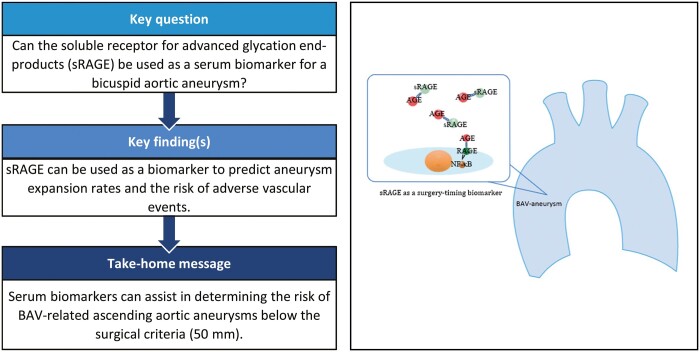

## INTRODUCTION

Bicuspid aortic valve (BAV) is the most common congenital heart abnormality with a prevalence of 1–2% in general [1]. Besides being a valvular disorder, it is associated with aortic dilatation and risk of aortic dissection and rupture. Studies on BAV have reported structural abnormalities of thoracic aortic tissue, including decreased collagen, elastin fragmentation, matrix disruption and smooth muscle cells (SMCs) apoptosis [2–4]. Although the current guidelines of American College of Cardiology/American Heart Association recommend the replacement of ascending aorta in patients with BAV if the ascending aortic diameter is >5.5 cm for non-Marfan patients or >4.5 cm for Marfan patients, decision-making regarding the management and timing of surgical intervention has no strong evidence yet [5]. Moreover, the pathogenesis of BAV with aneurysm is not clear, thereby impeding the personalization of treatment for aneurysm [6]. Therefore, identification of potential pathogenesis-related factors for early detection, determination of disease stages and progression of BAV aneurysm remains critically important [7]. In parallel, these markers may also serve as therapeutic targets in future [8, 9]. However, till date, reliable clinical circulating biomarker with screening, monitoring or predicting capabilities for disease-dependent pathological processes in BAV aneurysm is absent.

Recently, growing evidence from human studies have suggested a potential role of soluble receptor for advanced glycation end products (sRAGE) as a biomarker for vascular diseases, namely atherosclerosis and coronary artery disease [10, 11]. Advanced glycation end products (AGEs) constitute a heterogeneous group of irreversible products resulting from non-enzymatic glycation and oxidation of proteins, nucleic acids and lipids [12]. There are 2 isoforms of c-truncated receptor of advanced glycation end product (RAGE), namely sRAGE and endogenous secretory RAGE [13], both acting as a decoy for RAGE ligands and competing with membrane-bound RAGE for ligand binding [14], thus attenuating the activation of nuclear-factor kappa-B (NF-κB) pathway that can promote chronic inflammation of the aortic wall [15], and regulate MMP transcription [16, 17]. In human and animal experiments, inhibition of NF-κB activation can prevent the development of aortic aneurysm [18, 19]. Based on the existing literature, we hypothesized that plasma levels of sRAGE may differentiate between patients with BAV and the general population [20, 21], and more importantly, monitor the formation of ascending aorta aneurysm in patients with BAV.

## MATERIALS AND METHODS

### Study subjects

The study was approved by the Zhongshan Hospital Research Ethics Committee, and written informed consent was obtained from each patient. From March 2018 to January 2019, 64 patients, who would receive aortic valve replacement and/or ascending aorta replacement, were included in this study. Inclusion criterion required the age range to be 18–70 years, and selection was done considering bicuspid or tricuspid aortic valves (TAVs), valve function and origin of aneurysm. Exclusion criteria included active endocarditis, active malignancy, children-octogenarians and acute aortic dissection. Among the studied population, 44 individuals with BAV aneurysm were divided into 3 subgroups according to the diameter of ascending aorta: 4.0–4.5 cm in BAV 4.0–4.5 group (9 cases), >4.5 and < 5.0 cm in BAV 4.5–5.0 group (15 cases), >5.0 cm in BAV >5.0 group (20 cases). The TAV group comprised of the remaining 20 patients who had normal-size ascending aorta.

### Enzyme-linked immunosorbent assay

The protein levels of sRAGE in the serum were measured using an enzyme-linked immunosorbent assay kit (R&D Systems, Minneapolis, MN, USA) according to the manufacturer’s instructions.

### RNA isolation and reverse transcription

Total tissue RNA was isolated using TRIzol reagent (Invitrogen, NY, USA) according to the manufacturer’s instructions. cDNA was prepared from 1 mg of total RNA using a cDNA synthesis kit (Promega, Madison, WI, USA). Quantitative reverse transcription-PCR was conducted with SYBR Supermix (TAKARA). Expression of each target mRNA was calculated relative to that of glyceraldehyde-3-phosphate dehydrogenase (GAPDH) based on the threshold cycle (CT), as r = 2−Δ (ΔCT).

### Immunohistochemistry analysis

Aortic tissues were fixed in 4% paraformaldehyde for 24 h and embedded in paraffin. They were cut into 4-mm sections, placed in the blocking solution made of 0.5% BSA for 30 min and incubated with anti-rabbit IV collagen (Abcam, Cambridge, MA, USA ) antibody thereafter. The immune complex was visualized with the Dako REAL™EnVision™ Detection System, Peroxidase/DAB+, Rabbit/Mouse (Dako, USA/DEN), according to the manufacturer’s procedure. The nuclei were counterstained with haematoxylin and finally observed under a microscope (Nikon, Japan).

### Immunofluorescence staining

Serial 3-µm frozen sections of paired smooth muscle α-actin and cell apoptosis kit were rehydrated. After blocking the sections, they were incubated for 1 h at room temperature (20°C) with primary antibodies (Abcam, Cambridge, MA, USA) diluted in 1.5% BSA in PBS. Tissues were washed with PBS, fixed with 4% paraformaldehyde, and permeabilized with 0.1% Triton X-100. After the cells had been incubated with BSA for 30 min, primary antibodies were added overnight at 4°C, and incubated with Alexa Fluor 555-conjugated (Jackson Immuno Research) secondary antibodies for 1 h at room temperature in the dark, before counterstaining with 4ʹ,6-diamidino-2-phenylindole (DAPI; Sigma-Aldrich). Fluorescence signals were observed under the fluorescence microscope (Zeiss, Munich, Germany).

### Western blotting analysis

Total protein was extracted from aorta and then quantified using a BCA protein assay kit (Pierce, Rockford, USA). Tissue lysates were separated by SDS-PAGE (10%). After incubation with blocking buffer (TBS + 0.1% Tween-20 + 5% not fat milk), membranes were probed with primary antibodies overnight at 4°C, and incubated with primary antibodies against NF-κBP65 and GAPDH (Cell Signaling Technology, Inc.), and subsequently with an appropriate secondary antibody conjugated with horseradish peroxidase. Complexes were detected by chemiluminescence (Cell Signaling, Danvers, Co, USA).

### Transcriptome sequencing

Twelve tissue samples from patients with TAV/BAV (TAV, 3 cases; BAV 4.0–4.5 group, 3 cases; BAV 4.5–5.0 group, 3 cases; BAV > 5.0 group, 3 cases) were collected for transcriptome sequencing. Detailed experimental steps are in Supplementary Material.

### Primary human aorta smooth muscle cell extraction and treatment

We obtained an aortic sample from an ascending aortic replacement operation in a BAV patient and used this aortic tissue to extract the primary human aortic smooth muscle cells (HASMCs). Detailed experimental steps are in Supplementary Material.

HASMCs were then treated with AGE-BSA (Abcam, Cambridge, MA, USA) for 7 days at different concentrations (0, 1, 10, 20, 100 and 200 μg/ml).

### Age-treated human aortic smooth muscle cells in RNA isolation and reverse transcription

Different concentration gradients AGE-treated HASMCs total RNA was isolated by TRIzol reagent (Invitrogen, NY, USA). Subsequent cDNA preparation and RT-PCR procedures were consistent with those described above.

### Age-treated human aortic smooth muscle cells in western blotting analysis

Total protein was extracted from HASMCs by RIPA (Aspen, China) with Protease Inhibitor Cocktail (Roche, Basel, Swiss). The western blot procedure was the same as described previously. Primary antibodies against NF-κBP65, GAPDH (Cell Signaling Technology, Danvers, Co, USA) were also incubated on the membranes.

### Data analysis

All the values are expressed as mean ± standard deviation (SD). Continuous variables are represented as mean ± SD; they were compared across the 4 groups by one-way ANOVA (normality and homogeneity of variance were tested by Shapiro–Wilk W test and Variance Ratio test), followed by the multiple comparisons test (Bonferroni correction). The χ^2^ (≤20% expected value <5) and Fisher’s exact tests (>20% expected value <5) were used to compare qualitative variables across the groups of study subjects. Tests were 2-tailed, and values with *P* < 0.05 were considered statistically significant. All the statistic criteria are prespecified. Statistical analysis was performed with IBM SPSS Statistics 20 (SPSS, Inc., Chicago, IL, USA).

## RESULTS

### Baseline clinical characteristics

The demographic and clinical characteristics of patients in the 4 groups were comparable. Moreover, the 4 groups were well matched, except for the phenotype of aortic valves and diameter of ascending aorta (Table 1).

**Table 1:** Demographics of the studied population

	Control TAV (*n*=20)	BAV 4-4.5 (*n*=9)	BAV 4.5-5 (*n*=15)	BAV > 5 (*n*=20)	*P*-value
Gender (male/%)	14 (70.0)	5 (55.6)	10 (66.7)	14 (70.0)	0.995^a^
Age (year)	42.1 ± 6.1	47.1 ± 9.3	45.3 ± 7.1	45.7 ± 5.5	0.709
Mean diameter	33.9 ± 2.6	42.1 ± 1.6	46.8 ± 1.7	56.3 ± 6.2	0.000
Body weight (kg)	65.4 ± 6.0	64.8 ± 10.6	69.53 ± 7.3	65.9 ± 5.5	0.952
Smoking (*n*/%)	4 (20.0)	2 (22.2)	0 (0)	5 (25.0)	0.246^b^
Hypertension (*n*/%)	3 (15.0)	1 (11.1)	2 (13.3)	4 (20.0)	0.925^b^
Hyperlipidaemia (*n*/%)	1 (5.0)	0 (0)	1 (6.7)	0 (0)	0.634^b^
Cerebral vessel disease (*n*/%)	1 (5.0)	0 (0)	1 (6.7)	1 (5.0)	0.907^b^
Periphery vessel disease (*n*/%)	1 (5.0)	1 (11.1)	0 (0)	2 (10.0)	0.611^b^
Diabetic history (*n*/%)	1 (5.0)	3 (33.3)	2 (13.3)	3 (15.0)	0.256^b^
Aortic stenosis (*n*/%)	1 (5.0)	1 (11.1)	4 (26.7)	5 (25.0)	0.258^a^
Aortic regurgitation (*n*/%)	1 (5.0)	8 (88.9)	13 (86.7)	16 (80.0)	0.002^a^
Aortic valve replacement (*n*/%)	0 (0)	6 (66.7)	11 (73.3)	13 (65.0)	0.007^b^

a Means this type of data compared by χ^2^ tests.

b Means this type of data compared by Fisher’s exact tests. Data are shown by mean ± SD.

BAV: bicuspid aortic valve; TAV: tricuspid aortic valve.

### Serum levels of soluble receptor for advanced glycation end product in patients with bicuspid aortic valve and aortic aneurysm

**Figure 1:**
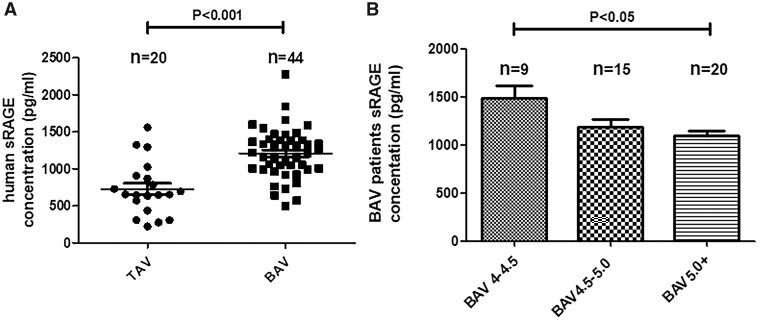
Serum levels of sRAGE in studied patients were examined by enzyme-linked immunosorbent assay. BAV: bicuspid aortic valve; TAV: tricuspid aortic valve. Data are shown as mean ± SD (independent experiments). (**A**) Serum sRAGE levels in TAV group (*n* = 20) and BAV group (*n* = 44). Dots represent serum levels in each patient. (**B**) Serum sRAGE levels in BAV groups [BAV 4.0–4.5 group (*n* = 9); BAV 4.5–5.0 group (*n* = 15); BAV >5.0 group (*n* = 20)]. sRAGE: soluble receptor for advanced glycation end product.

Serum level of sRAGE in the BAV groups was remarkably higher than in the control TAV group (*P* < 0.001). As in Fig. 1B, we found the serum sRAGE levels to tend to decrease with the diameter of ascending aorta in patients with BAV, especially when the diameter was >5.0 cm. We hypothesized that there was a statistical difference in serum sRAGE levels among BAV groups, and Bonferroni correction was performed at α = 0.05 level. There was a statistical difference between BAV 4.0–4.5 and BAV >5.0 groups (1.36-folds, *P* < 0.01), and there was no statistical difference between BAV 4.0–4.5 and BAV 4.5–5.0 groups or between BAV 4.5–5.0 and BAV > 5.0 groups. The serum levels of sRAGE in control TAV subjects and patients with BAV are summarized in Fig. 1.

### Transcript expression levels of nuclear-factor kappa-B signalling pathway

**Figure 2:**
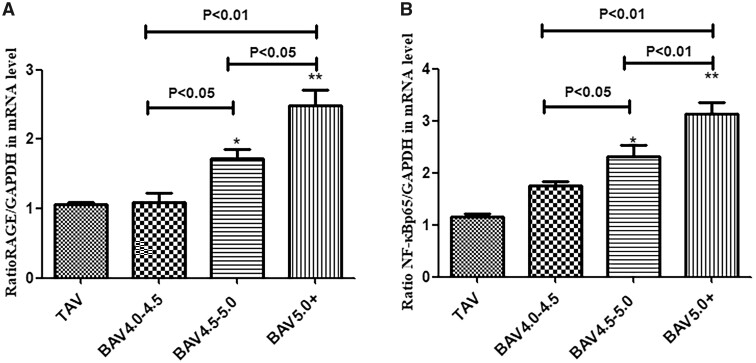
The mRNA expression levels of RAGE and NF-κBp65 in different patients were detected by quantitative reverse transcription-PCR. Glyceraldehyde-3-phosphate dehydrogenase is shown as the loading control. BAV: bicuspid aortic valve; TAV: tricuspid aortic valve. Data are shown as mean ± SD. *P < 0.05 vs control TAV, **P < 0.01 vs control TAV. (**A**) Ratio of RAGE to glyceraldehyde-3-phosphate dehydrogenase in TAV and BAV groups. (**B**) Ratio of NF-κBp65 to glyceraldehyde-3-phosphate dehydrogenase in TAV and BAV groups. RAGE: receptor of advanced glycation end product.

Results of quantitative reverse transcription-PCR showed that compared to the values in patients with the TAV, transcript expression levels of RAGE (BAV 4–4.5 group: 1.24-fold, *P* < 0.05; BAV 4.5–5 group: 1.53-fold, *P* < 0.01; BAV > 5 group: 2.19-fold, *P* < 0.01), and NF-κBp65 mRNA (BAV 4–4.5 group: 1.63-fold, *P* < 0.05; BAV 4.5–5 group: 1.97-fold, *P* < 0.01; BAV > 5 group: 2.46-fold, *P* < 0.01) were significantly increased in the 3 BAV groups. This phenomenon was more notable in the BAV >5 group. Transcript expression levels of the important genes in NF-κB signalling pathway in the studied population are summarized in Fig. 2.

### Matrix disruption in bicuspid aortic aneurysm

**Figure 3:**
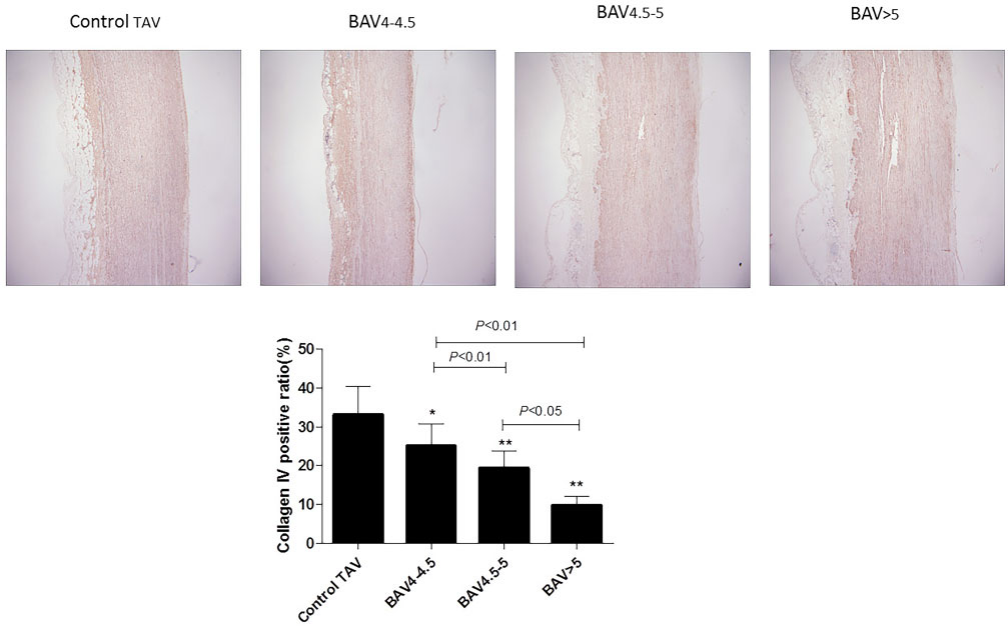
Collagen IV protein expression levels in different patients were detected by immunohistochemistry. The groups were as follows: Control TAV, BAV 4–4.5, BAV 4.5–5, BAV >5. Data are shown as mean ± SD representing at least 3 independent experiments. *P < 0.05 vs control TAV, **P < 0.01 vs control TAV. BAV: bicuspid aortic valve; TAV: tricuspid aortic valve.

A semi-quantitative analysis showed that collagen IV immunostaining in ascending aorta of the control TAV group was significantly higher than that in the 3 BAV groups (BAV 4–4.5 group: 0.76-fold, *P* < 0.05; BAV 4.5–5 group: 0.53-fold, *P* < 0.01; BAV >5 group: 0.24-fold, *P* < 0.01). The positive rate of collagen IV in BAV >5 group was the lowest among the 3 BAV groups (*P* < 0.01). Collagen IV immunostaining was notably reduced in BAV 4.5–5 group compared to that in the BAV4–4.5 group. Figure 3 shows the representative immunostaining of collagen IV in ascending aorta of the studied patients.

### Smooth muscle cells apoptosis in bicuspid aortic aneurysm

**Figure 4:**
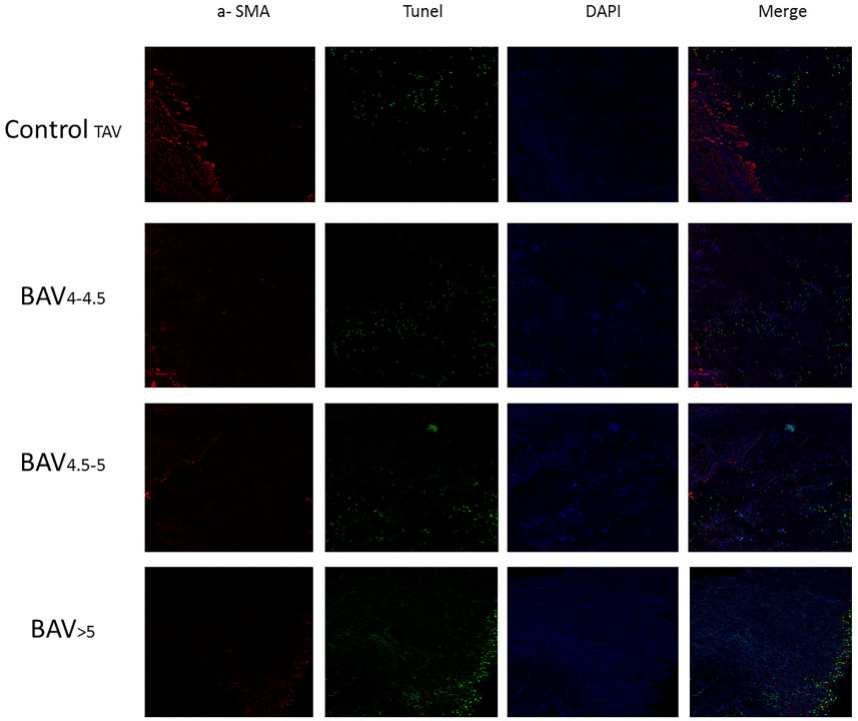
The smooth muscle α-actin protein and TUNEL in different patients were detected by confocal microscope. The groups were as follows: Control TAV, BAV 4–4.5, BAV 4.5–5, BAV >5. The representative immunofluorescence showed that only a small number of apoptotic cells were found in the control TAV group, while they were increased in the BAV groups. Number of apoptotic cells in the BAV >5 group was highest among the 3 BAV groups. BAV: bicuspid aortic valve; TAV: tricuspid aortic valve.

To further confirm the above findings regarding the remodelling of aorta in patients with BAV, we tested the apoptosis of SMCs in ascending aorta by dual staining for smooth muscle α-actin and TUNEL. The results revealed that only a small number of apoptotic cells were found in the control TAV group, while they were remarkably increased in the BAV groups. Among the 3 BAV groups, the number of apoptotic cells in the BAV >5 group was remarkably higher than those in the rest (Fig. 4).

### Nuclear-factor kappa-B signalling pathway activation

**Figure 5:**
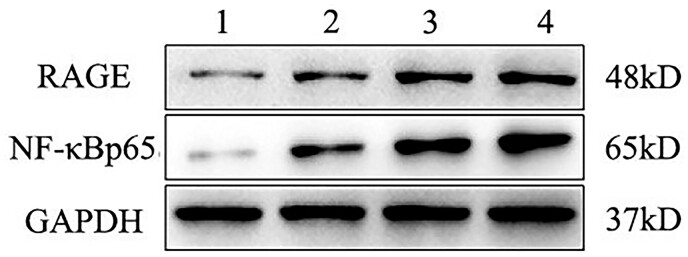
The protein levels of RAGE and NF-κBp65 in different patients were analysed by western blotting. Glyceraldehyde-3-phosphate dehydrogenase protein is shown as the loading control. 1 represents the TAV group; 2 represents the BAV 4.0–4.5 group; 3 represents the BAV 4.5–5.0 group; 4 represents the BAV >5.0 group. BAV: bicuspid aortic valve; GAPDH: glyceraldehyde-3-phosphate dehydrogenase; RAGE: receptor of advanced glycation end product; TAV: tricuspid aortic valve.

Compared to the control TAV group, protein expression of RAGE (BAV 4–4.5 group: 1.82-fold, *P* < 0.05; BAV 4.5–5 group: 2.49-fold, *P* < 0.01; BAV >5 group: 3.96-fold, *P* < 0.01), and NF-κBp65 RAGE (BAV 4–4.5 group: 2.04-fold, *P* < 0.01; BAV 4.5–5 group: 3.19-fold, *P* < 0.01; BAV >5 group: 7.36-fold, *P* < 0.01) was remarkably increased in the 3 BAV groups; activation of RAGE/NF-κB pathway was more prominent in the BAV >5 group. Therefore, we concluded that RAGE/NF-κB pathway was activated as ascending aortic aneurysm developed in patients with BAV. The data are shown in Fig. 5.

### Age/receptor of advanced glycation end products downstream pathways activation

**Figure 6:**
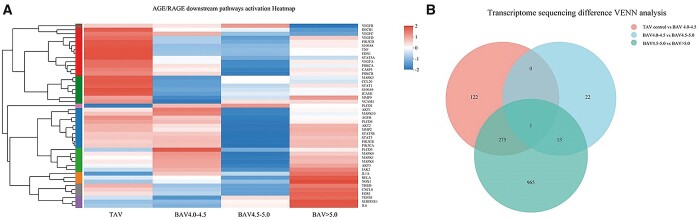
In transcriptome sequencing, differences existed between TAV group and BAV group, and such differences were also found within BAV groups. (**A**) AGE/RAGE-downstream-related transcriptome sequencing heatmap. (**B**) Transcriptome sequencing differences VENN analysis. AGE: advanced glycation end products; BAV: bicuspid aortic valve; RAGE: receptor of advanced glycation end product; TAV: tricuspid aortic valve.

After transcriptome sequencing of 12 ascending aorta tissue samples, a series of gene transcriptional differences between TAV group and BAV groups was obtained. Using KEGG (https://www.kegg.jp/dbget-bin/www_bget?AGE/RAGE related molecular pathways presented in map04933) they were further analysed, and relative differences across the groups were found, as presented in the form of heat map in Fig. 6. In this sequencing, we found a potential research site for the aetiology of BAV through VENN algorithm, and a gene in the antigen-recognition region of immunoglobulin—IGLV8-61 gene transcription showed significant statistical differences across each group (TAV group vs BAV 4.0–4.5 group; BAV 4.0–4.5 group vs BAV 4.5–5.0 group; BAV 4.5–5.0 group vs BAV >5.0 group). Whether this gene locus is involved in the pathogenesis of BAV or it is just a statistical error caused by a relatively small sample size remains to be further studied.

### Effect of AGE-BSA treatment on transcription and translation in human aortic smooth muscle cells

**Figure 7:**
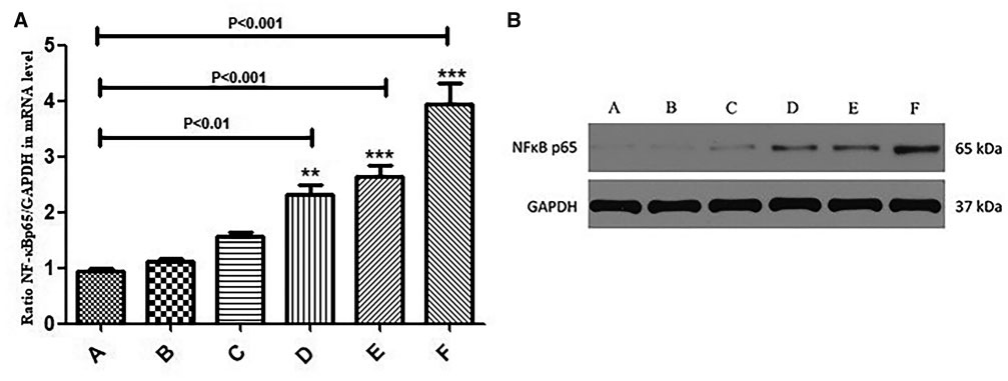
The mRNA and protein expression levels of NF-κBp65 in human aortic smooth muscle cells for different gradient treatments, as detected by quantitative reverse transcription-PCR and western blot. Glyceraldehyde-3-phosphate dehydrogenase is shown as the loading control. (A) 0 μg/ml AGE-BSA; (B) 1 μg/ml AGE-BSA; (C) 10 μg/ml AGE-BSA; (D) 20 μg/ml AGE-BSA; (E) 100 μg/ml AGE-BSA; (F) 200 μg/ml AGE-BSA. Data are shown as mean ± SD. **P < 0.01 vs A group, ***P < 0.001 vs A group. (**A**) Ratio of NF-κBp65 to glyceraldehyde-3-phosphate dehydrogenase mRNA levels in 6 AGE-BSA treatments groups. (**B**) The protein levels of NF-κBp65 in 6 AGE-BSA treatments groups.

Based on the transcription and translation of NF-κBp65 in aortic tissues, we studied the mRNA and protein expression differences of NF-κBp65 after AGE-BSA gradient treatments for 7 days. As shown in Fig. 7, with the increase of AGE-BSA concentration, the expression of NF-κBp65 tended to be up-regulated.

## DISCUSSION

This is a first exploratory study to investigate the relationship between serum sRAGE levels and BAV aneurysm, and our results found that serum levels of sRAGE were higher in patients with BAV aneurysm than in those with the control TAV group and although, in comparison between groups of BAV, not every group was statistically different, we still found that serum sRAGE levels tended to decreased as the diameter of ascending aorta in patients with BAV. In previous experiments, inhibition of NF-κB activation can prevent the development of aortic aneurysm [18, 19]. Previous studies found an NF-κB binding site within the MMP promoter is known to regulate the MMP gene expression that possesses the unique ability to degrade elastin and collagen [22], and NF-κB signalling pathway is important for cells to respond to numerous extracellular signals [23]. Our study also showed that RAGE/NF-κB pathway is activated with the development of ascending aortic aneurysm, including collagen fragmentation and SMCs apoptosis, in patients with BAV. In subsequent cytological experiments, we demonstrated the abnormal activation of this pathway under high concentrations of AGE-BSA treatment. The pathogenesis of aneurysm has been studied extensively. Historically, BAV-related aneurysm pathogenesis had been attributed to the destruction of collagen and elastin in media and adventitia, and loss of SMCs with thinning of aortic wall [2–4]. Previous studies had reported that AGEs interact with receptors for AGEs (RAGE) and activate NF-κB, increasing gene expression and releasing inflammatory cytokines (IL-1β, IL-2, IL-6 and TNF-α), thereby generating reactive oxygen species [24–26].

In the present study, we found an inverse correlation between sRAGE and collagen IV content of the aortic wall of patients with BAV and aneurysm. More importantly, our data also indicated the cellular and extracellular processes underlying aortic enlargement to be quite serious in the normal-sized or slightly dilated BAV aorta (mean diameter 4.2 cm). Evidence of a ‘preclinical’ aortopathy may add to the current debates on whether and how the ascending aorta at the time of surgery for BAV stenosis or regurgitation can be concomitantly addressed. Although this is currently the gold standard, routine computed tomography angiography scanning could not provide enough information for surgeons to understand the fundamental aortopathy at cellular level [27]. Therefore, the optimal timing of aortic surgery in patients with thoracic aortic aneurysm remains uncertain for both BAV and TAV cases.

Since echocardiography is widely available, economical, sensitive and effective in identifying aortic aneurysms, using sRAGE merely as a biomarker may not yield additional significant prognostic value. However, sRAGE biomarker may be very useful to predict aneurysm expansion rates and the risk of future ruptures. It may offer a significant benefit in the stratification and monitoring of patients with small yet, fast-growing BAV aneurysm and high risk of dissection or rupture. Considering the lack of information on the risk of aortopathies from common imaging techniques, our new findings hint at exploration of sRAGE as a potential biomarker to predict the presence, progression and prognosis of BAV aneurysm that would be especially important for patients with high risk [28].

Although the number of subjects (44 BAV cases and 20 matched controls) enrolled into this study was relatively small, results from this analysis are encouraging us to pursue larger cohort studies to construct an effective prediction model for the severity of BAV aneurysm. Another limitation of this study is that it was a single-centre study, a multicentre study can further validate the clinical value of our results.

## CONCLUSION

sRAGE has potential to be a biomarker for BAV-related ascending aortic aneurysm, and its serum levels can be associated with the structural disorder of extracellular tissues of the ascending aortic walls and abnormal signalling pathway of the cells in the cases of ascending aortic aneurysms. Moreover, we also verified this idea *in vitro* cytological experiments. These findings will help provide more effective information for surgical intervention of ascending aortic aneurysms.

## SUPPLEMENTARY MATERIAL

Supplementary material is available at *ICVTS* online.

## Funding 

This work is supported by the National Natural Science Foundation of China (Grant Number 81671942), Y.S.

**Conflict of interest:** none declared.

## AUTHOR CONTRIBUTIONS

**Hao Jia:** Methodology; Writing—original draft. **Le Kang:** Methodology; Writing—review & editing. **Shuyang Lu:** Methodology; Writing—review & editing. **Zhenhang Chen:** Formal analysis. **Jinqiang Shen:** Formal analysis. **Ben Huang:** Methodology. **Yunzeng Zou:** Conceptualization. **Yongxin Sun:** Conceptualization; Funding acquisition.

## REVIEWER INFORMATION

Interactive CardioVascular and Thoracic Surgery thanks Luca Di Marco, Thomas Schachner and the other anonymous reviewers for their contribution to the peer review process of this article.

## Supplementary Material

ivab242_Supplementary_Data

## ABBREVIATIONS

CAD: coronary artery disease
AGE: Advanced glycation end products
BAV: Bicuspid aortic valve
HASMCs: Human aortic smooth muscle cells
NF-κB: Nuclear-factor kappa
BRAGE: Receptor of advanced glycation end products
SMCs: Smooth muscle cells
sRAGE: Soluble receptor for advanced glycation end products
TAV: Tricuspid aortic valve
