# Supplementary material for: Circulating soluble receptor of advanced glycation end product is associated with bicuspid aortic aneurysm progression via NF-κB pathway
Source: Interact Cardiovasc Thorac Surg. 2021 Oct 14;34(2):274–82. doi: 10.1093/icvts/ivab242 (PMC8766214; doi:10.1093/icvts/ivab242)
Supplement: ivab242_Supplementary_Data [file ivab242_supplementary_data.docx]

**Supplementary material**

**Method:**

**2.7. Transcriptome sequencing**

Total RNA was extracted from the tissue using TRIzol® reagent (Plant RNA Purification Reagent for plant tissue), according to the manufacturer’s instructions (Invitrogen), and genomic DNA was removed using DNase I (TaKaRa). RNA quality was determined by 2100 Bioanalyser (Agilent) and the same quantified using the NanoDrop 2000 (NanoDrop Technologies). Only high-quality RNA samples (OD260/280=1.8–2.2, OD260/230 ≥ 2.0, RIN ≥ 6.5, 28S:18S ≥ 1.0, > 1 μg) were used for the construction of sequencing library. RNA-seq transcriptome library was prepared following TruSeqTM RNA sample preparation kit instructions, from Illumina (San Diego, CA), using 1 μg of total RNA. Briefly, mRNA was isolated according to polyA selection method using oligo(dT) beads and then fragmented by fragmentation buffer. Next, double-stranded cDNA was synthesised using a SuperScript double-stranded cDNA synthesis kit (Invitrogen, CA) with random hexamer primers (Illumina). Then the synthesized cDNA was subjected to end-repair, phosphorylation, and ‘A’ base addition, according to Illumina’s library construction protocol. Libraries were size-selected for cDNA target fragments of 300 bp on 2% Low Range Ultra Agarose, followed by PCR amplification using Phusion DNA polymerase (NEB) over 15 PCR cycles. After quantification by TBS380, paired-end RNA-seq sequencing library was sequenced with the Illumina HiSeq X Ten/NovaSeq 6000 sequencer (2 × 150bp read-length). Clean reads were separately aligned to the reference genome with orientation mode using HISAT2 software.

**2.8. Primary human aorta smooth muscle cell extraction and treatment**

We applied fetal bovine serum (Gibco, 10099141C) adherent method to 30 pieces (0.2cm*0.2cm) of shredded ascending aortic tissue. After 12 hours, we turned over the cell flask, and took the excess serum out and then added smooth muscle cell medium (ScienCell Research Laoratories, San Diego, CA) into the cell flask. Primary human aortic smooth muscle cells (HASMCs) were harvested after 3 weeks. These cells were digested with trypsin in the logarithmic growth stage and prepared into cell suspensions with a concentration of 1 × 10^5^ /ml. The HASMCs were cultured in CO2 (5%) incubator at 37 ℃ for 24 h to allow them to stick to the wall, and remain serum-free for 24 h thereafter. After culture, half of the cell samples was replaced by drug-containing medium, whereas the control group was replaced by solvent-containing medium.
